# Implant Infectious Diseases: An Introduction to Biomaterials for ID Physicians

**DOI:** 10.1093/ofid/ofaf411

**Published:** 2025-07-12

**Authors:** Lauren E Kemp, Alexander M Tatara

**Affiliations:** Division of Infectious Diseases and Geographic Medicine, The University of Texas Southwestern Medical Center, Dallas, Texas, USA; Division of Infectious Diseases and Geographic Medicine, The University of Texas Southwestern Medical Center, Dallas, Texas, USA; Department of Biomedical Engineering, The University of Texas Southwestern Medical Center, Dallas, Texas, USA

**Keywords:** biofilm, biomaterials, device-associated infection, local drug delivery, *Staphylococcus aureus*

## Abstract

Implanted biomedical devices are becoming ubiquitous in the practice of medicine but are at risk for biofilm-related infection. The biomaterial composition of these devices can significantly affect their risk for infection. Biomaterials design is complex with compositional choices leading to different properties, including mechanical strength, biodegradation rate, and ability to locally release therapeutics. In this review, we introduce the field of “Implant Infectious Diseases,” review practical biomaterial fundamentals for the infectious disease clinician, and apply these principles to case vignettes. This review serves as a primer for a broad infectious disease audience to better understand the role of biomaterials in medical devices and as therapeutics.

## Teaser

Implanted biomaterials have become ubiquitous in the practice of medicine and are vulnerable to infection. In this review, we introduce the “Implant ID” specialist and discuss basic principles of biomaterials to an ID audience with accessible vignettes and without technical jargon.

The science of biomaterials is rapidly advancing. Inventions such as cardiac implantable electronic devices, prosthetic joints, and insulin pumps are improving and/or saving lives around the world. Biomaterials are now also used as drug delivery vehicles and were recently leveraged to develop mRNA vaccines as a rapid response to the COVID-19 pandemic [1]. In addition to new vaccine technology, biomaterials have benefitted the field of infectious diseases (ID) with new ways to deliver antibiotics and treat infected wounds [2–4].

However, biomaterials can also be vulnerable to infection by tenacious biofilm-producing organisms. Once a mature biofilm has been established on a biomaterial surface, cure by antibiotics alone is difficult and often requires surgical intervention. These device-related infections are a large burden on both patients and the healthcare system [5–14] (Table 1). Within ID, subspecialty areas of focus have developed, such as solid organ transplantation, oncology, and HIV ID specialists. As the use of biomaterials (and their complications) continues to increase, this may drive the creation of “implant ID” specialists, experts with additional special interest in preventing, diagnosing, and managing device infections.

**Table 1.** Examples of Device-related Infections With Representative Device Biomaterial Composition and Estimates of Incidence per Year in the United States

Infection	Example Biomaterial	Estimated Infections Per Year
Prosthetic valve endocarditis [5]	Pyrolytic carbon (ceramic)	3240
Cardiac implantable electronic device infection [6, 7]	Poly(urethane) (polymer)	8000
Vascular graft infection [8]	Poly(ethylene terephthalate) (polymer)	2000–16 000
Left ventricular assist device driveline infection [9, 10]	Poly(tetrafluoroethylene) (polymer)	480
Periprosthetic joint infection [11]	Titanium alloy (metal)	40 000
Breast implant infection [12]	Poly(dimethylsiloxane) (polymer)	12 500
Central line-associated bloodstream infection [13]	Poly(urethane) (polymer)	92 000
Ventriculoperitoneal shunt infection [14]	Poly(dimethylsiloxane) (polymer)	2880
Catheter-associated urinary tract infection [13]	Poly(vinyl chloride) (polymer)	449 000

To better understand implant infections, it is helpful to have some background knowledge on biomaterials. This review is targeted toward ID clinicians to serve as an introduction to the basic principles of biomaterials without using technical jargon or requiring advanced knowledge of chemical syntheses. We then apply these lessons to several ID-related biomaterial vignettes as examples of how to apply biomaterial principles to infectious problems.

## PRINCIPLES OF BIOMATERIALS

**Figure 1.**
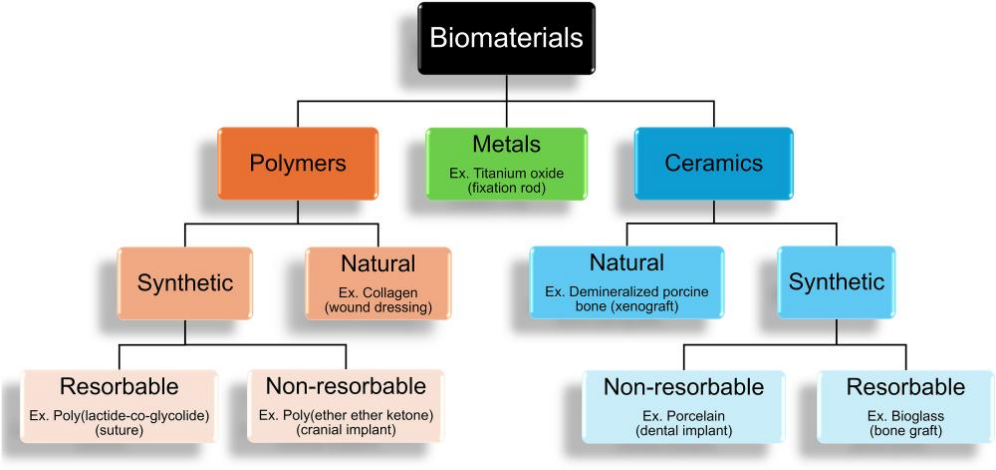
Classification scheme for biomaterials with example devices in parentheses.

**Figure 2.**
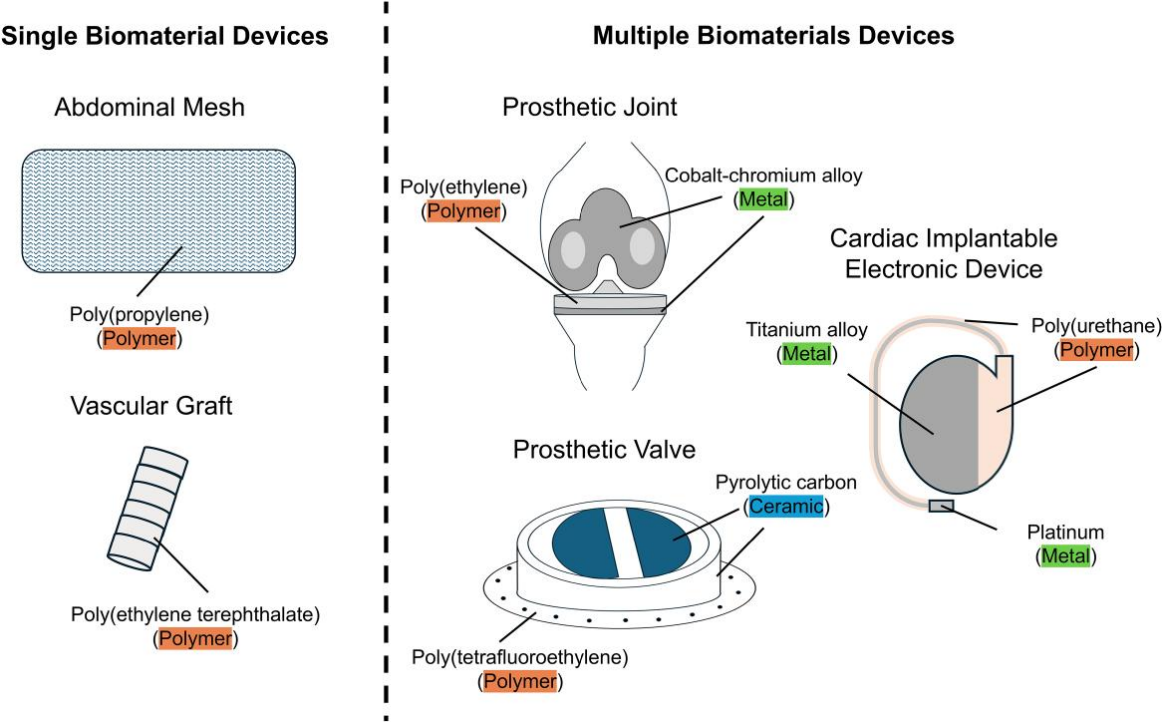
Examples of devices fabricated from a single type of biomaterial (left) versus multiple types of biomaterials (right).

Biomaterials are materials that can be implanted in the body and elicit a desired physiologic response. These can be intended for temporary use, such as a peripheral vascular catheter, or permanent implantation, such as a prosthetic heart valve. The 3 major compositional types of biomaterials are polymers, ceramics, and metals (Figure 1). Devices can be fabricated from a single biomaterial or by combining any number of different biomaterials (Figure 2). Understanding which biomaterials are synthetic and the timescale of their resorption/replacement with native tissue is particularly important for ID physicians given the increased infection risk of foreign bodies.

### Polymers

Polymers are chains of repeating subunits (monomers). Proteins, for example, are polymers made up of amino acids. Polymers can be naturally derived or synthetically fabricated. Although it is not within our scope to review every clinically translated polymer, we will discuss the properties of natural versus synthetic polymers and give several ID-related examples of each.

Naturally derived polymers have the advantage of inherent motifs that allow host cell adhesion as well as mechanisms for host-mediated degradation such as enzymatically cleavable sequences. However, naturally derived polymers may also have motifs recognized by the immune system and induce unwanted inflammation. Naturally derived polymers may also carry some risk of disease transmission depending on how they were collected and processed. Examples of devices composed of naturally derived polymers include wound dressings [15], meshes for hernia repair [16], and hemostatic glue [17]. Collagen is a classic example of a natural polymer and is the most prevalent structural protein in the body [18]. It is available in many clinical applications, such as wound dressings for diabetic foot ulcers [15] and drug delivery for osteogenic growth factors [19]. Collagen is rapidly degraded by collagenases. As a hydrogel, collagen can absorb aqueous solutions and has been used as a “sponge” to load and locally release bioactive molecules such as antibiotics and growth factors. In a meta-analysis of randomized controlled trials (RCTs), collagen sponges loaded with gentamicin significantly reduced surgical site infections although trials for diabetic foot infection were less convincing [20, 21]. Naturally derived polymers do not need to be sourced from mammalian proteins; for example, alginate (seaweed) [22], chitosan (shellfish and fungi) [23], and cellulose (plants) [24] are all biomaterials in current clinical products for wound treatment.

Synthetic polymers are commonly used in implanted devices such as breast implants, urinary and vascular catheters, and polymer bone cement. Unlike naturally derived biomaterials, synthetic biomaterials may or may not be biodegradable. Although lack of degradation can be a disadvantage regarding infection risk, there are devices such as vascular grafts where degradation could be catastrophic. Synthetic polymers can be designed to have a wide variety of physicochemical properties, including tunable mechanical strength and degradation rate. However, they do not inherently contain motifs allowing for specific host cell interactions, such as adhesion ligands or enzymatically cleavable sites; these elements need to specifically be programmed into their structure if desired. An example of a widely used biodegradable synthetic biomaterial is poly(lactide-co-glycolide) (PLGA), a copolymer of glycolic acid and lactic acid. Devices incorporating PLGA include biodegradable sutures [25], long-acting depots for hormone agonist delivery [26], and antibiotic-releasing microparticles to treat dental infection [4]. By altering the ratio of glycolic to lactic acid, the polymer can be programmed to biodegrade at different rates; this allows tuning of properties such as how rapidly sutures resorb or how quickly antibiotic is released. PLGA has also been used in implantable depot vaccines [27, 28] with a recent clinical study demonstrating efficacy as an immunotherapy for metastatic melanoma [29].

Polymers are the most versatile class of biomaterials and have been successfully translated into a variety of medical implant applications [30, 31]. Due to their diversity, it is difficult to draw any broad conclusions on infection risk and should be evaluated on a device-by-device basis. In general, biomaterials that degrade and are replaced by vascularized host tissue are likely at less risk of infection than nonbiodegradable materials.

### Ceramics

Ceramics are rigid mineral-based biomaterials and are typically strong and hard. Given their mechanical properties, ceramic biomaterials are most often applied in orthopedic and craniofacial applications.

Bone and teeth, for example, are composed of the natural ceramic hydroxyapatite (HA), a crystal structure including calcium, phosphate, and hydroxide. HA is used as a bone graft and can be autologous AKA “autograft” (harvested from another site in the patient's body such as the iliac crest), allograft (derived from cadavers), or xenograft (derived from animal sources). Under physiologic conditions, HA does not degrade; it requires osteoclast-mediated resorption for remodeling. In addition to its uses as a graft, HA can be coated onto other materials to encourage osteointegration. In 1 RCT, patients undergoing tibial distraction osteogenesis received either typical titanium pins or stainless-steel pins coated with HA to promote osteointegration [32]. Nine (39.1%) of 23 patients in the control group and 1/23 (4.3%) patient in the HA-coated group developed infection/loosening (*P* < .001), which may have been due to improved osteointegration with the adjacent host tissue. However, when a similar RCT was conducted in 40 patients for wrist fixation, there were no significant differences in infection rates [33].

There may be applications where more rapid biomaterial degradation is advantageous. Beta-tricalcium phosphate (BTCP) is a synthetic ceramic that resorbs more quickly than HA [34, 35]. In a small RCT, patients undergoing surgical repair of bony defects were treated with either autograft or BTCP and showed similar fusion rates between the groups without significant differences in infection [36]. Meta-analyses have suggested that BTCP is associated with similar or lower infection rates than HA for spinal surgery and fractures [37, 38]. Composites combining HA and BTCP can also be synthesized to take advantage of properties from both types of ceramics (burst of early resorption and release of calcium and phosphate ions with a long-lasting scaffold for bony deposition). In 1 small RCT, this composite material was associated with fewer infections than autograft for treatment of acute fractures [39].

In addition to calcium-based ceramics, silicone-based ceramics have also been translated for clinical use. Bioactive glass or “bioglass” was invented as a material designed to adhere to bone and promote HA deposition in vivo [40]. Although it has been primarily used in dental applications, small RCTs have shown equivalence to allograft for bony defects [41, 42]. There is also evidence that suggests bioglass has inherent antimicrobial properties, possibly from local pH and osmotic pressure changes at the material surface [43]. In a nonrandomized trial of 116 patients treated for chronic osteomyelitis with bioactive glass as part of their graft material (in addition to antibiotic therapy), cure rates were reported as 90%, although this was difficult to interpret given no control cohort [44]. Compared to other ceramics, bioglass is limited due to its brittle nature but can be considered for non-load-bearing applications [45]. Silicone-based biomaterials are currently being explored in preclinical models as implantable vaccine platforms to prevent device-related infection [46, 47].

In current clinical practice, autologous bone graft has been the gold standard for ceramic biomaterials. Autograft has inherent growth factors and contains populations of progenitor cells which may aid in wound healing [48]. However, the donor site for autograft is at risk for infectious complications. Using “off-the-shelf” synthetic ceramics mitigates donor-site morbidity. Compared to HA, BTCP and other calcium-based ceramics can be synthesized with shorter resorption times that may decrease risk of infection from replacement of foreign material with native tissue [39]. Bioactive glass is a resorbable silicone-based ceramic that may have inherent antimicrobial properties and merits further well-designed trials [45].

Ceramics are also applied as endovascular biomaterials. Prosthetic heart valves are subdivided into bioprosthetic/tissue valves (derived from xeno- or allografts) or mechanical valves that are entirely synthetic. Mechanical valves can have different components depending on design, such as discs, ball/cages, hinges, and a sewing ring. These components can be synthesized from polymers, metals, or ceramics depending on biomechanical need and biocompatibility. Low-temperature isotropic pyrolytic carbon is a strong ceramic that somewhat resists clot formation and mechanical wear, making it an attractive biomaterial for heart valves [49]. It was a major component of the original 1968 DeBakey-Surgitool mechanical valve and continues to be used in current mechanical valves [49, 50]. Adherence of bacteria to pyrolytic carbon valves is modulated by surface free energy and roughness, suggesting that bioceramic design may be able to impact risk of attachment for some species like *Staphylococcus epidermidis* [51]. Interestingly, data suggest that mechanical valves are less likely to have infectious relapse than bioprosthetic valves when used to replace native tissues in the setting of endocarditis although these are largely from meta-analyses and may be biased as older patients are more likely to receive bioprosthetic valves [52, 53].

### Metals

Metals are most used in dental, orthopedic, and cardiac applications because of their stiff and strong mechanical properties. Although there are metals that can be engineered to biodegrade [54], the majority used in clinical practice are nonresorbable. Metallic biomaterials have been engineered to promote integration with host tissue. For example, while stainless steel can have stronger biomechanical properties, titanium alloys have superior ingrowth of bone tissue resulting in lower rates of clinical failure [55, 56].

In vitro, staphylococci are better able to adhere to representative ceramics (hydroxyapatite) and polymers (polymethyl methacrylate) than metal (stainless steel and titanium alloy) [57, 58]. In small prospective clinical studies, fixation devices made from titanium alloys were significantly less likely to result in severe infection and had decreased biofilm compared to stainless steel [59, 60]. This has also been demonstrated in animal studies, although pathogen virulence factors may play a more significant role than any specific type of metal [61, 62]. Among clinical data, 1 retrospective study examined ∼98 000 hip arthroplasty cases from a registry categorized by type of biomaterial articulation surfaces (metal on metal, metal on ceramic, metal on polymer, ceramic on ceramic, or ceramic on polymer) and found that periprosthetic joint infection rates were lowest in those with ceramic-on-ceramic devices [63]. However, the authors were unable to account for host factors such as baseline comorbidities or body mass index, which may have confounded results. In a subsequent meta-analysis of hip arthroplasties including 11 RCTs, there was no significant differences in periprosthetic joint infection risk and type of articulating biomaterial [64].

Cardiac implantable electronic devices (CIED) include a titanium metal pulse generator connected to leads coated in polymers (such as poly[urethane]) ending in platinum metal electrodes. When directly comparing titanium and poly(urethane), the metal surfaces had decreased bacterial burden [65]. Prior scientific statements from the American Heart Association on CIED infections discussed the in vitro data suggesting different CIED biomaterials had different propensities for bacterial adherence [66]; however, this has not been evaluated in clinical CIED studies, and the most recent scientific statement no longer discusses the impact of biomaterial selection [67].

In addition to orthopedic and vascular application, metals are sometimes used in biliary and ureteral stents. There is speculation that metal stents may be more susceptible to infection or encrustation compared to polymer stents in this application but there are no comparative studies to confirm this [68].

In general, biomaterials are composed of polymers, ceramics, metals, or combinations of those classes. Ceramics and metals are relatively strong and stiff, whereas polymers have a wider variety of mechanical properties and applications. Biomaterials may be derived from natural products or synthetically fabricated; properties including ability to biodegrade and/or deliver therapeutics rely on the composition of the biomaterial.

## INFECTIOUS DISEASES BIOMATERIAL CASE VIGNETTES

### To Bead or Not to Bead: Differences in Biomaterial Properties Affect Decision-making

Biomaterial elution for local delivery of antibiotics to sites of bone and joint infection has been explored since the 1970s [69]. An advantage of this approach is that high local concentrations of antibiotics such as aminoglycosides can be achieved with low systemic levels to avoid adverse effects. Poly(methyl methacrylate) (PMMA), also known as bone cement, is used in arthroplasty to fix prostheses into place. It is a nonbiodegradable polymer that can be synthesized in the operating room and molded in situ. During its synthesis, antibiotics can be incorporated into the final PMMA device. This has been applied in cement used to fix replacement joints during revision, “spacers” used during 2-stage revision of periprosthetic joint infection, and as beads that can be placed in infected bony defects for local release [3].

Understanding the basic principles of biomaterials allows us to predict some of the limitations with PMMA as a drug delivery vehicle. PMMA is nonbiodegradable, which is a useful property for permanently fixing orthopedic devices into place. However, the majority of incorporated antibiotic remains locked within the polymer matrix and cannot be released into tissues. The antibiotic that can be released is generally adsorbed to its surface and released rapidly within several days [70]. Beads are more effective than spacers in antibiotic release due to a higher surface area to volume ratio. Because PMMA is nonbiodegradable, beads are often surgically removed following drug release or otherwise can act as an additional nidus of infection [71, 72]. Although there is a scarcity of well-controlled RCTs surrounding antibiotic-eluting PMMA, some meta-analyses suggest benefit [73, 74].

**Figure 3.**
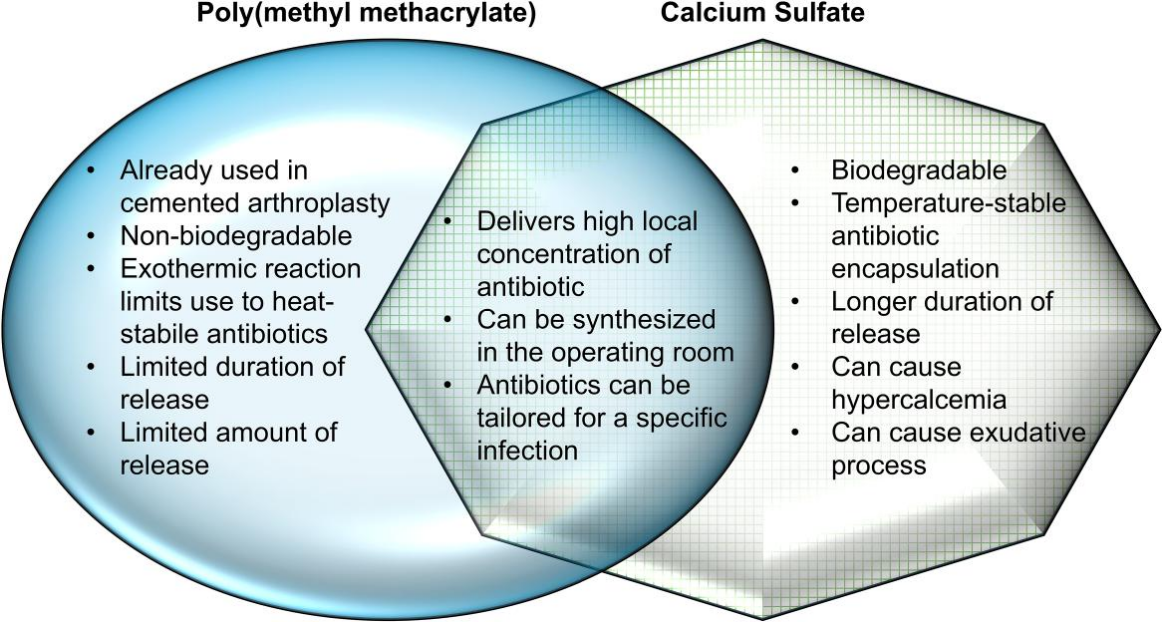
Venn diagram summarizing the advantages and disadvantages of poly(methyl methacrylate) and calcium sulfate biomaterial beads for orthopaedic applications.

Given the limitations of PMMA, alternative vehicles for antibiotic delivery have been developed. Calcium sulfate is a biodegradable ceramic that can be prepared in beads and loaded with antibiotics. Unlike PMMA, calcium sulfate beads fully degrade in an aqueous environment. Calcium sulfate-based beads resorb in 1–3 months, although resorption time is likely variable due to amount of biomaterial, size and vascularization of wound, and other host factors [75]. Theoretically, calcium sulfate should have a longer and greater release of antibiotics compared to PMMA as it can completely biodegrade. However, in vitro studies showed antibiotic-loaded calcium sulfate performing only slightly better than PMMA at early timepoints [76]; some studies report a greater duration of inhibitory effect, whereas others demonstrate relatively similar duration of effectiveness [77, 78]. In a small RCT comparing calcium sulfate to PMMA beads in patients with chronic osteomyelitis, infection eradication was similar but those in the PMMA group required significantly more procedures [79]. One of the drawbacks of calcium sulfate beads is hypercalcemia; in 1 study of 755 patients where calcium sulfate beads were used in arthroplasty, 41 (5.4%) patients developed hypercalcemia including 2 that were symptomatic [80]. The degradation and release of calcium and sulfate ions can also cause a large exudate and wound drainage, theorized to be due to hyperosmolarity and/or an inflammatory response. It appears to occur less often with smaller volumes [80, 81]. By understanding biomaterial properties, we can better understand the different advantages and disadvantages of antibiotic bead systems for orthopaedic applications (Figure 3). Although the decision on which biomaterial to apply is often made by the orthopedic surgery team, collaborative multidisciplinary models of care have led to increased conversations between ID physicians and surgeons who seek input on the best strategies for local treatment of complex musculoskeletal infections [82].

### Pushing the Envelope: An Anti-infective Biomaterial Life Cycle

**Figure 4.**
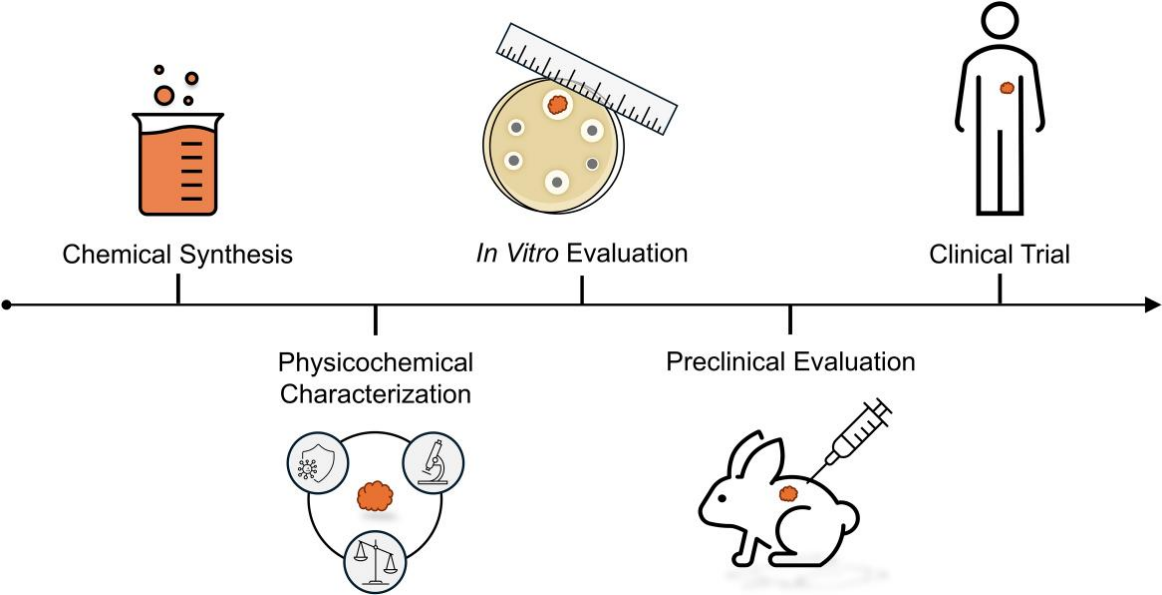
Development cycle of a biomaterial-based device to prevent or treat infection.

This vignette illustrates how a new biomaterial goes through conceptualization, in vitro characterization, preclinical modeling, and, last, clinical trials for an ID application (Figure 4). Many biodegradable synthetic polymers degrade into acidic byproducts such as lactic acid, which may inhibit wound healing [83, 84]. In an effort to design biomaterials that degrade into milder byproducts, amino acids have been explored in unconventional combinations. However, most amino acid-derived polymers have been limited by unfavorable immunogenicity and mechanical properties [84]. Biomaterial scientists found that tyrosine can be used to generate monomers of desaminotyrosyl-tyrosine alkyl ester. These monomers can then be used as building blocks to synthesize biodegradable polymers such as polycarbonates [85]. In physiologic conditions, polycarbonates degrade into tyrosine and desaminotyrosyl, a compound found in olives. The mechanical properties and hydrophobicity of these polymers can be controlled by altering the length of the alkyl ester chain [86]. Different polymer “envelopes” have been synthesized that release antibiotics for approximately one week and biodegrade over several months [87].

CIEDs such as pacemakers and defibrillators are predominantly metal with polymer-insulated leads. Both metal and polymer components are vulnerable to infection with rates of 0.1%–8.6% [88]. Tyrosine-derived, polymer-based envelopes were loaded with rifampin and minocycline and used to protect CIEDs. In a rabbit study, pacing devices were implanted alone or within the polymer pouch and the pockets were intentionally inoculated with *S epidermidis*. The polymer pouch resulted in a sterile pocket in 2/3 animals with only a few colonies in the remaining animal [89].

In the WRAP-IT RCT, approximately 7000 patients were randomized to receive a typical cardiac implantable electronic device or the device enclosed in a tyrosine-derived polymer envelope impregnated with minocycline and rifampin [2]. The antibiotic-releasing polymer envelope was safe and significantly decreased rates of major CIED infection (hazard ratio, 0.6; 95% confidence interval, .36–.98; *P* = .04) with a mean follow up of 21 months. In a subgroup analysis, the envelope was particularly effective against staphylococci infection with a 76% reduction (*P* = .01) [90]. Use of biomaterial antibiotic-eluting envelopes is now encouraged in high-risk patients by the American Heart Association [67]. This vignette demonstrates how a new biomaterial can be imagined, synthesized, characterized, and eventually reach patients to prevent morbid infections related to medical implants.

### Should I Stay or Should I Go: Device Removal Decisions and Biomaterials

A common dilemma posed to implant ID physicians: a patient recently had an abdominal mesh placed during hernia repair and now has a staphylococcal abscess abutting the mesh. If the abscess can be locally debrided and/or drained, does the patient require mesh replacement or can the device be salvaged? There is reasonable evidence that central lines, for example, can be salvaged in ∼80% of cases even when infected with biofilm-associated organisms such as *S epidermidis* [91]—what about other devices? Does device biomaterial composition impact odds of successful salvage?

Abdominal meshes can be fabricated from nonresorbable synthetic polymers such as poly(propylene) (Figure 2), resorbable synthetic polymers such as poly(glycolic acid), or resorbable naturally derived polymers (“biologic mesh”) such as collagen originating from decellularized porcine submucosa. Meta-analyses suggest that resorbable meshes trend toward lower infection risk even in contaminated fields, although statistically biomaterials alone have not been proven to modify risk, likely because of small sample size [92, 93]. In retrospective studies of polymer-based mesh, salvage was significantly most successful with poly(propylene) meshes compared to poly(tetrafluoroethylene) (PTFE) meshes [94, 95]. Composition was not the only biomaterial factor; fabrication parameters were important as lightweight poly(propylene) with large pore sizes had significantly less relapse rates than mid- or heavyweight poly(propylene). Ultimately, even in the most successful group (lightweight and/or macroporous poly[propylene]), salvage was successful in only 62.5%–72% of cases [94, 95]. Biomaterials derived from natural tissues do not always equate with lower infection risks. Biologic meshes have not been shown to have improved salvage rates compared to macroporous poly(ethylene); in fact, synthetic meshes were associated with fewer surgical site infections than biologic meshes, hypothesized to be due to increased vascularization and possible bacterial adhesin motifs on biologics as seen in animal studies [96, 97].

Although replacing an abdominal mesh is more difficult than a central line, surgery for other implanted devices can be even more difficult when infected. For example, surgery for vascular graft infection is fraught with challenges. Vascular grafts can be derived from allograft or autograft vessels or “prosthetic,” ie, fabricated from synthetic nonresorbable polymers such as poly(ethylene terephthalate) (PET) or expanded PTFE (Figure 2). Microbial adherence and biofilm production to graft polymers is a complex interaction between the biomaterial, adsorbed host proteins, and bacteria. The molecular interactions between biomaterials and biofilm have been recently reviewed [98, 99]. Specific to prosthetic vascular grafts, bacteria bind and produce biofilm more easily on PET compared to PTFE in vitro [100, 101]. However, when the 2 biomaterials are coated with human plasma before bacterial exposure, binding increases to PTFE and decreases to PET [101]. Clinically, early studies suggested PTFE was more resistant to infection than PET but a more recent meta-analysis showed no differences in infection rates [102]. Likewise, no antimicrobial coating has been demonstrated to prevent polymer graft infection [103]. On the other hand, the luminal coatings of vascular grafts used to promote host integration can impact infection; prospectively (although not randomized) investigators found that polymer grafts coated with collagen had higher infection rates than those coated with gelatin [104].

Overall, synthetic vascular grafts are more prone to infection than naturally derived grafts. In a canine model, animals had a thoracoabdominal aortic graft placed using either a human-derived xenograft or a synthetic PTFE graft. Following *Staphylococcus aureus* bacteremia, two thirds of the polymer grafts were infected and none of the animals with human-derived grafts had infection [105]. Likewise, a meta-analysis suggested that patients treated with excision and graft replacement have higher reinfection rates with polymer-based grafts, even if the polymer releases antibiotic locally [106]. In terms of treatment of infected vascular grafts, the most recent scientific statement by the American Heart Association recommends graft excision when infection is caused by *Pseudomonas,* methicillin-resistant *S aureus,* or other multidrug-resistant organisms [107, 108]. Other factors affecting likelihood of salvage include the degree of anastomotic disruption and time of infection from initial graft placement; there are not enough high-quality data to guide this decision based on biomaterial type [107]. Following excision and need for reimplantation, experts suggest rifampin-bonded or silver-coated polymer grafts for patients who would not tolerate the longer surgeries required for autografts/allografts but acknowledge that there are not randomized prospective data in this area.

Regardless of type of device, some patients will have an infected device that cannot be removed because of anatomy, unlikelihood of surviving additional surgery, or other factors. The duration of therapy and the use of continued antibiotic suppression following a therapeutic period is a complicated question that often benefits from multidisciplinary discussion and careful risk/benefit discussions with patients [109]. Persistent antibiotic usage does invoke resistance, and the long-term detriments of suppressive therapy on the microbiome remain unclear. For individual types of device infection, such as periprosthetic joint infection and spinal hardware infection, there are large retrospective datasets that do not show a clear benefit in suppression, especially after 1 year of therapy [110, 111]. There are new data for monitoring modalities such as positron emission tomography/computed tomography that may help guide the duration of antibiotic suppression [112]. Across different types of devices, experts have noted a paucity of high-quality prospective comparative studies to understand the efficacy of suppressive antibiotic therapy, and this remains an active area of research in implant ID [109, 111–113].

### Taking COVID Down a PEG: Biomaterials for Immunoengineering

The COVID-19 pandemic was estimated to cause 15 million deaths worldwide by the World Health Organization [114]. At the start of the pandemic, there were no available vaccines or treatments, and biomaterials scientists mobilized to create novel antiviral strategies [115].

Before COVID-19, virologists learned how to design respiratory surface glycoproteins as stable and immunogenic antigens that facilitated rapid adaption of the SARS-CoV-2 spike protein as a vaccine target [116]. Many conventional infectious vaccines are protein based. These often require complex manufacturing processes, intricate supply chain logistics, and are difficult to modify. In recent years, mRNA has been explored to stimulate host cells to produce protein antigens of interest in situ. The process of creating mRNA is similar regardless of sequence, streamlining manufacturing and storage. If mutations result in the need for different epitope targets, the mRNA sequence in question can be easily modified. Once delivered, mRNA carries additional advantages, including its rapid degradation in the cytosol following translation and lack of need for nuclear translocation.

However, delivery of mRNA-based antigen is challenging. As a charged and complex macromolecule, mRNA cannot penetrate cells and is rapidly enzymatically degraded by serum RNAses [117]. Before COVID-19, biomaterial scientists had designed different drug delivery vehicles to protect mRNA cargo and facilitate cellular delivery for immunoengineering purposes [47]. Immunoengineers were able to design cationic lipids and lipid-based nanoparticles to better carry negatively charged mRNA [118]. As lipid-based vehicles, the particles can fuse with host cell envelopes to deliver the mRNA intracellularly. To prolong circulation and prevent premature mRNA clearance, immunoengineers added poly(ethylene glycol) (PEG) moieties to the lipid nanoparticles [119]. These advances by biomaterials scientists significantly contributed to mRNA COVID-19 vaccine development which is estimated to have saved more than 1.5 million lives [1]. Lipid nanoparticle mRNA delivery systems are currently in clinical trials for other infectious diseases and cancers [120].

## CONCLUSIONS

Advances in biomaterial science have led to a plethora of implantable devices. These devices have different risk profiles for infection based on properties such as ease of bacterial adhesion, surface structure, and ability to degrade and be replaced by host tissue. Biomaterials can also be programmed to release antibiotics and even function as vaccine platforms to train the immune system against specific pathogens. A basic understanding of biomaterials, biomaterial properties, and their function can help ID clinicians assist in diagnostic and therapeutic decision-making surrounding patients with implant-associated infections. Future clinical studies designed to better understand the impact of biomaterial choices in medical device infection are needed to improve decision-making in Implant ID.

## Notes

***Acknowledgments.*** The authors thank Dr. Hayden Andrews for thoughtful comments on an earlier draft. This information has not been previously presented at any meetings.

***Author Contributions.*** Conceptualization, A.M.T.; writing, L.E.K. and A.M.T.; editing, L.E.K. and A.M.T.

***Financial support.*** We acknowledge NIH/NIAID K08 AI180362 (A.M.T.).
